# The Supramolecular Chemistry of Cycloparaphenylenes and Their Analogs

**DOI:** 10.3389/fchem.2019.00668

**Published:** 2019-10-09

**Authors:** Dapeng Lu, Qiang Huang, Shengda Wang, Jinyi Wang, Pingsen Huang, Pingwu Du

**Affiliations:** ^1^School of Pharmacy, Anhui Medical University, Hefei, China; ^2^Hefei National Laboratory for Physical Sciences at the Microscale, iChEM (Collaborative Innovation Center of Chemistry for Energy Materials), CAS Key Laboratory of Materials for Energy Conversion, Department of Materials Science and Engineering, University of Science and Technology of China, Hefei, China

**Keywords:** supramolecular chemistry, fullerene guest, non-fullerene guest, carbon nanohoop, cycloparaphenylene

## Abstract

Cycloparaphenylenes (CPPs) and their analogs have recently attracted much attention due to their aesthetical structures and optoelectronic properties with radial π-conjugation systems. The past 10 years have witnessed a remarkable advancement in CPPs research, from synthetic methodology to optoelectronic investigations. In this present minireview, we highlight the supramolecular chemistry of CPPs and their analogs, mainly focusing on the size-selective encapsulation of fullerenes, endohedral metallofullerenes, and small molecules by these hoop-shaped macrocycles. We will also discuss the assembly of molecular bearings using some belt-persistent tubular cycloarylene molecules and fullerenes, photoinduced electron transfer properties in supramolecular systems containing carbon nanohoop hosts and fullerene guests, as well as the shape recognition properties for structure self-sorting by using dumbbell-shaped dimer of [60]fullerene ligand. Besides, the supramolecular complexes with guest molecules other than fullerenes, such as CPPs themselves, iodine, pyridinium cations, and bowl-shaped corannulene, are also discussed.

## Introduction

**Figure 1:**
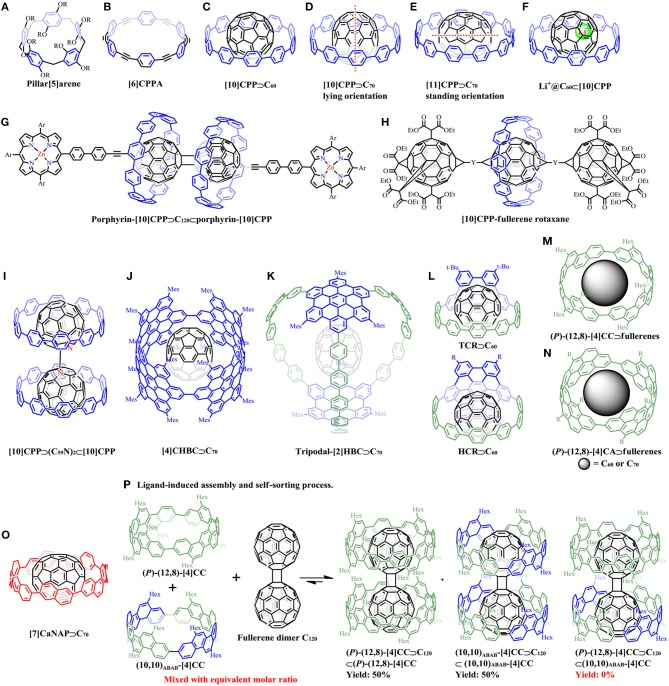
Examples of pillar[n]arenes, CPPAs and supramolecular complexes of carbon nanohoops with fullerenes: **(A)** The structure of pillar[5]arene. **(B)** The structure of [6]CPPA. **(C)** The supramolecular structure of [10]CPP⊃C_60_. **(D)** [10]CPP⊃C_70_ in its “lying” orientation with its long axis perpendicular to [10]CPP plane. **(E)** [11]CPP⊃C_70_ in its “standing” orientations with its long axis within the [11]CPP plane. **(F)** Li^+^@C_60_⊂[10]CPP. **(G)** The 2:1 complex of porphyrin-[10]CPP and fullerene dimer: porphyrin-[10]CPP⊃C_120_⊂porphyrin-[10]CPP. **(H)** [10]CPP-fullerene rotaxane. **(I)** [10]CPP⊃(C_59_N)_2_⊂[10]CPP complex. **(J)** [4]CHBC⊃C_70_. **(K)** tripodal-[2]HBC⊃C_70_. **(L)** TCR⊃C_60_ and HCR⊃C_60_. **(M)** (*P*)-(12,8)-[4]CC⊃C_60_ or C_70_. **(N)** The π-lengthened version of (*P*)-(12,8)-[4]CA⊃C_60_ or C_70_. **(O)** [7]CaNAP⊃C_70_. **(P)** The ligand-induced self-sorting process for two diastereomers.

Supramolecular chemistry is the subject of the association of two or more chemical species held together by intermolecular forces, such as electrostatic interactions, hydrogen bonding, van der Waals forces, etc., which could lead to organized entities of higher complexity (Lehn, 1985, 1988). It is one of today's fastest growing disciplines, crossing a range of subjects from biological chemistry to materials science, and shows great potential in the fields of catalysis, drug delivery, biotherapy, electrochemical sensor, self-healing materials (Zhang and Wang, 2011; Yan et al., 2012; Dong et al., 2015; Yang et al., 2015; Zhang et al., 2017a; Zhou et al., 2017). As one of the most important aspect of supramolecular chemistry, the host-guest molecular recognition requires that the two species must complement each other both in geometry (size and shape) and binding sites (Lehn, 1985, 1988). Macrocyclic structures, in principle, meet the requirements as they usually contain the cavities, clefts, and pockets with appropriate size and shape that provide the framework for substrate species by multiple non-covalent interactions. The representative macrocyclic molecules during the development of supramolecular chemistry, such as crown ether, cyclodextrins, calixarenes, and cucurbiturils, have been the classical structures in this field (Yang et al., 2015; Zhou et al., 2017). Recently, the introduction of pillar[*n*]arenes (Figure 1A) as new types of macrocyclic hosts by Ogoshi et al. (2008), rapidly received significant attention for their prominent host-guest properties.

Meanwhile, another type of carbon-rich macrocyclic molecules with radially oriented π systems pointing inwards to the cavity have emerged as a new class of strained, non-planar aromatic structures, which were named as cycloparaphenylenes (CPPs) or carbon nanohoops because of their structural relationship with carbon nanotubes (CNTs) (Jasti et al., 2008; Jasti and Bertozzi, 2010). Despite their simple structures, however, the synthesis of CPPs was only achieved in 2008 from curved molecular precursors after intensive efforts (Jasti et al., 2008) Following this work, several other novel strategies for CPP synthesis were developed and a number of CPP-related carbon nanorings with various sizes and atomic compositions were prepared (Darzi et al., 2015; Segawa et al., 2016). More importantly, Itami et al. reported the successful synthesis of a carbon nanobelt, [12]carbon nanobelt ([12]CNB) comprising a closed loop of fully fused edge-sharing benzene rings in 2017 (Povie et al., 2017). Furthermore, development of this synthetic strategy to the preparation of [16]CNB and [24]CNB analogs were also reported by the same group (Povie et al., 2018). Using a new ligand system, the yield of the final belt-forming, nickel-mediated reaction for [12]CNB was improved from 1 to 7%, and [16]CNB and [24]CNB were obtained in 6 and 2% yield, respectively. These studies are important steps toward the bottom-up synthesis of other carbon nanobelt structures and CNTs. Another interesting and valuable work which should be mentioned is the thermally induced cycloreversion strategy for the synthesis of carbon nanohoops reported by Huang et al. (2016). They converted the anthracene photodimer synthon into anthracene-incorporated aromatic macrocycle through ring expansion reaction based on the cycloreversion of its dianthracene core. This work sheds light on the utility of the anthracene photodimerization-cycloreversion method for “bottom-up” carbon nanohoop synthesis. The past 10 years have witnessed a remarkable advancement in CPPs research, from synthetic methodology to optoelectronic investigations due to their size-dependent behavior and promising applications in materials (Segawa et al., 2012; Wu et al., 2018; Huang et al., 2019; Toyota and Tsurumaki, 2019; Xu and Delius, 2019).

In a recent work, Delius et al. overviewed the host-guest chemistry of carbon nanohoops, the preparation of mechanically interlocked architectures, and crystal engineering (Xu and Delius, 2019). In this present minireview, we only highlight the supramolecular chemistry of CPPs and their analogs, mainly focusing on the size-selective encapsulation of fullerenes, endohedral metallofullerenes, and small molecules by these hoop-shaped macrocycles. We will also discuss the assembly of molecular bearings using some belt-persistent tubular cycloarylene molecules and fullerenes, photoinduced electron transfer properties in supramolecular systems containing carbon nanohoop hosts and fullerene guests, as well as the shape recognition properties for structure self-sorting by using dumbbell-shaped dimer of [60]fullerene ligand. Besides, the supramolecular complexes with guest molecules other than fullerenes, such as CPPs themselves, iodine, pyridinium cations, and bowl-shaped corannulene, are also discussed.

## Supramolecular Complexes Consisting of CPP_s_ and Fullerenes

The first series of macrocyclic hosts was the molecules with sp^2^/sp-hybridized carbon atoms, cyclic paraphenyleneacetylenes (CPPAs) (Figure 1B), reported by Kawase et al. (1996). The complexation between CPPA congeners and fullerenes were extensively studied (Kawase et al., 2003a,b, 2007; Miki et al., 2013). Although CPPA derivatives tend to form tight complexes with C_60_, their unstable nature hindered further experimental studies. In contrast, the solely sp^2^-hybridized CPP derivatives without acetylene linkers are sufficiently stable, and could similarly encapsulate fullerene molecules.

The initial example of the host-guest complex of this type was reported by Iwamoto et al. (2011). The CPP receptor with 10 phenylene units ([10]CPP) has an ideal diameter (1.38 nm) to accommodate C_60_ (0.71 nm) (Figure 1C), showing a binding constant *K*_a_ of 2.79 × 10^6^ M^−1^ in toluene determined by fluorescence quenching titration, which was two orders of magnitude higher than those obtained for [6]CPPA⊃C_60_ (Kawase et al., 2003a). The variable-temperature NMR (VT-NMR) spectroscopy experiments showed that the rapid exchange between free [10]CPP and [10]CPP⊃C_60_ took place at room temperature, and the energy barrier for the exchange was determined to be 59 kJmol^−1^. The crystal structure of [10]CPP⊃C_60_ obtained by Jasti' group revealed the presence of convex-concave π-π interactions (Xia et al., 2012). It is noteworthy that C_60_ can be selectively encapsulated by [10]CPP among the mixture of [8]-[12]CPPs, indicating that the cavity sizes of other CPPs were not appropriate for constructing a strong complex with C_60_. Interestingly, it was found that C_70_, which has an ellipsoidal shape with long axis of 0.796 nm and short axis of 0.712 nm, could also be encapsulated by [10]CPP in its “lying” orientation with its long axis perpendicular to [10]CPP plane (Figure 1D), but with reduced association constant *K*_a_ (8.4 × 10^4^ M^−1^ in toluene) compared with [10]CPP⊃C_60_ (Iwamoto et al., 2013). Nevertheless, C_70_ was adopted the “standing” orientations to be accommodated in the cavity of [11]CPP with its long axis within the [11]CPP plane (Figure 1E). Besides, [11]CPP deformed into an ellipsoidal shape to maximize the van der Waals interactions with the long axis of C_70_. All these results indicated the size- and orientation selectivity for the CPP⊃fullerene systems. Furthermore, a deep exploration by analyzing geometry structures through theoretical calculations revealed that C_70_ selectively adopts lying, standing, and half-lying orientations when combined with [10]CPP, [11]CPP, and [12]CPP, respectively (Yuan et al., 2015).

In 2014, Shinohara et al. demonstrated the high binding abilities of [11]CPP toward C_82_-based endohedral metallofullerenes, including Gd@*C*_2v_-C_82_, Tm@*C*_2v_-C_82_, and Lu_2_@*C*_2v_-C_82_, which provided a facile non-chromatographic strategy for Gd@C_82_ extraction and enrichment from crude fullerene mixtures (Nakanishi et al., 2014). Later, another example of C_82_-based endohedral metallofullerene peapod, [11]CPP⊃La@C_82_ was reported (Iwamoto et al., 2014). The solid structure of the complex was determined by X-ray crystallographic analysis, which showed that the La atom was located near the periphery of [11]CPP rather than the tube axis with the dipole moment of La@C_82_ nearly perpendicular to the CPP axis. These evidence demonstrated the different orientations of La@C_82_ in CPP and CNT peapods, which suggests that the orientation of La@C_82_ in CNT was mainly determined by interactions among the adjacent ones. More importantly, due to the strong electron accepting properties of La@C_82_, partial charge transfer (CT) from [11]CPP to La@C_82_ in the ground state was firstly observed by electrochemical experiments combined with UV/Vis-near-infrared (NIR) titration studies and density functional theory (DFT) calculations, but no fully ionized complex was formed.

The CPP-based fully ionized complex, Li^+^@C_60_⊂[10]CPP, was synthesized and characterized by Ueno et al. (2015) (Figure 1F). The ionic crystal structure was confirmed by X-ray crystallographic analysis. Unlike the empty C_60_, the cationic Li^+^@C_60_ core drastically increased the electron accepting ability which could induce strong charge transfer from the electron donors. Cyclic voltammetry experiments revealed that Li^+^@C_60_ was harder to be reduced when accommodated by [10]CPP than Li^+^@C_60_ itself, which could be ascribed to the higher electron density around the Li^+^@C_60_ cage through CPP to Li^+^@C_60_ charge transfer interaction. The strong charge transfer interaction also caused the positive charge of the lithium cation delocalized to the outer CPP ring. The broadened absorption bands at around 350 nm and in the NIR region was also related to this interaction. Besides, photoluminescence (PL) lifetime of Li^+^@C_60_⊂[10]CPP (2.5 ns) is shorter than that of [10]CPP (4.3 ns) and C_60_⊂[10]CPP (4.3 ns), suggesting that the charge transfer (CT) interaction may occur.

Recently, Delius et al. reported the synthesis of a porphyrin-[10]CPP conjugate, in which [10]CPP moiety served as a supramolecular junction for charge transfer between a zinc porphyrin electron donor and fullerene electron acceptor (Xu et al., 2018b). Efficient photoinduced electron transfer was observed with a lifetime of charge separation state up to 0.5 μs in the 2:1 complex between [10]CPP and the fullerene dimer (Figure 1G). The intramolecular energy transfer between [10]CPP and porphyrin was also observed. Later, the same group achieved the synthesis of two [2]rotaxanes consisting of one [10]CPP moiety binding to a central fullerene with bis-adduct binding site and another two fullerene hexakis-adduct stoppers using a concave-convex π-π template strategy (Figure 1H) (Xu et al., 2018a). [10]CPP served as an effective supramolecular directing group with the central fullerene as an efficient convex template, steering the reaction exclusively toward two *trans* regioisomers in the final step. The mechanically interlocked structures of [2]rotaxanes were analyzed by variable-temperature NMR (VT-NMR) and mass spectrometry. Transient absorption spectra revealed the interesting consequences of the mechanical bond on charge transfer processes. A later work conducted by Wegner et al. used a dumbbell-shaped dimeric azafullerene [(C_59_N)_2_] as the ligand to combine with two [10]CPP rings, giving [10]CPP⊃(C_59_N)_2_⊂[10]CPP complex (Figure 1I) (Rio et al., 2018). Two stage binding constants were determined to be *K*_a1_ = 8.4 × 10^6^ M^−1^ and *K*_a2_ = 3.0 × 10^6^ M^−1^, respectively, with weak interactions between the two CPP rings. Photoinduced partial charge transfer was observed from [10]CPP to (C_59_N)_2_ by differential pulsed voltammetry experiments.

## Supramolecular complexes consisting of π-extended carbon nanohoops and fullerenes

As the π-π interaction operates *via* the surface-to-surface contacts in supramolecular chemistry, it becomes important for large aromatic moieties with increasing π-surface areas. Based on the rapid development of the synthesis strategies, carbon nanohoops with embedded polycyclic aromatic hydrocarbon (PAH) structures, such as hexa-*peri*-hexabenzocoronene (HBC) (Quernheim et al., 2015; Lu et al., 2016; Huang et al., 2019), were subsequently prepared. These π-extended macrocycles usually show larger binding constants with guest molecules due to their larger contact area compared with simple CPP hosts.

The [4]cyclo-2,11-*para*-hexa-*peri*-hexabenzocoronene ([4]CHBC) synthesized in our laboratory was found to selectively incorporate C_70_ with a binding constant *K*_a_ of 1.07 × 10^6^ M^−1^ in toluene (Figure 1J), but no evidence of complexation with C_60_ guest was observed, which could be due to the “standing” or “lying” orientations of C_70_ in the cavity of the carbon nanoring (Lu et al., 2017). Similarly, another HBC-containing three-dimensional capsule-like carbon nanocage, tripodal-[2]HBC also exhibited the preference of affinity toward C_70_ (*K*_a_ = 1.03 × 10^5^ M^−1^ in toluene) rather than C_60_, which was demonstrated by MS, NMR, and photophysical experiments (Figure 1K) (Cui et al., 2018). More recently, our group achieved the synthesis of two novel π-extended crown-like molecules (TCR and HCR) with embedded curved nanographene units, HBC or TBP (tribenzo[*fj,ij,rst*]pentaphene) (Huang et al., 2019). These two species were found to show high binding affinity toward guest molecule C_60_ with the association constants *K*_a_ of 3.34 × 10^6^ M^−1^ for TCR⊃C_60_, and 2.33 × 10^7^ M^−1^ for HCR⊃C_60_, respectively (Figure 1L). The gradual increasement in binding constants from [10]CPP⊃C_60_ (*K*_a_ = 2.79 × 10^6^ M^−1^) (Iwamoto et al., 2011) to TCR⊃C_60_, then HCR⊃C_60_, should be ascribed to the increasing π-surfaces that could provide stronger π-π interactions between the hosts and C_60_. Besides, photocurrents were generated when using these molecular crowns or their supramolecular complexes on FTO electrodes under visible light irradiation. Time-resolved spectroscopic measurements suggested fast photoinduced electron transfer in the supramolecular heterojunctions.

The recently reported shape-persistent tubular carbon nanorings demonstrated the binding ability with fullerenes. Five structural isomers of [4]cyclo-2,8-chrysenylene ([4]CC) (Hitosugi et al., 2011), which were named as (*P*)-(12,8)-, (*P*)-(11,9)-, (10,10)_AABB_-, (10,10)_ABAB_-, and (+)-(16,0)-[4]CC, can form 1:1 complex with C_60_ in solution (Isobe et al., 2013). The highest binding constant among similar complexes was recorded for (*P*)-(12,8)-[4]CC⊃C_60_ (Figure 1M) in *o*-DCB with *K*_a_ = 4.0 × 10^9^ M^−1^, while isomers of (*P*)-(11,9)-, (10,10)_AABB_-, and (10,10)_ABAB_-[4]CC also showed the binding constant above 10^9^ M^−1^. The lowest *K*_a_ was recorded for (+)-(16,0)-[4]CC⊃C_60_ (2.0 × 10^4^ M^−1^ in *o*-DCB), but was still higher than that for [10]CPP⊃C_60_ (6.0 × 10^3^ M^−1^ in *o*-DCB) (Iwamoto et al., 2011). These results clearly show that the belt-persistency in tubular structures also plays a crucial role in binding with fullerenes besides the cavity size. Therefore, a molecular rolling bearing with C_60_ in the [4]CC bearing was constructed as the bearing can hold the fullerene molecule tightly to prevent its run-out motion. The C_60_ molecule did not exchange and took rapid relative rolling motion on the NMR timescale within the bearing from the ^1^H NMR analysis of (*P*)-(12,8)-[4]CC⊃C_60_. The crystal structures of this molecular bearing was further analyzed by X-ray diffraction, demonstrating the presence of smoothly curved surface that allows the dynamic motion of C_60_ even in the solid state (Sato et al., 2014). Theoretical studies by density functional theory (DFT) indicates that the calculated association energies were quite method-dependent, and the energy barriers for the rolling motions within the bearing were as low as 2–3 kcal mol^−1^ with two distinct rolling motions (precession and spin) (Isobe et al., 2015).

Besides C_60_ guest, another twelve fullerenes, including C_70_, nine exohedral functionalized fullerenes, and two endohedral fullerenes, were selected and assessed as rolling journals in the belt-persistent [4]CC bearing (Hitosugi et al., 2013). [4]CC tolerated the modified fullerenes but with reduced binding constant. C_70_ was found to be superior guest not only for the high binding constant (*K*_a_ = 5.0 × 10^9^ M^−1^ in DCB), but also for its tolerance of introduction of bulky shaft without obvious decrease in binding constant. A lengthened version of (*P*)-(12,8)-[4]cyclo-2,8-anthanthrenylene ((*P*)-(12,8)-[4]CA) can also bind with C_60_ and C_70_ (Figure 1N) with enhanced association enthalpy as the increase of the C-C contact area compared with the shorter congener (*P*)-(12,8)-[4]CC (Matsuno et al., 2013, 2015).

The electronic properties of the molecular bearings were then systematically studied. The bearing systems can generate charge-separated species under light irradiation. (*P*)-(12,8)-[4]CC⊃C_60_ system exhibits a rapid back electron transfer to give triplet C_60_ journal after the formation of triplet charge-separated species *via* photoinduced electron-transfer (Hitosugi et al., 2014). The lengthened version of [4]CA⊃C_60_ could generate a triplet excited state at the outer bearing, whereas the endohedral fullerene Li^+^@C_60_ enabled the back electron transfer processes without triplet excited species (Hitosugi et al., 2015).

Although there existed tight association between (*P*)-(12,8)-[4]CC and C_60_, the solid-state dynamic rotations of C_60_ still enabled reorientation by a small energy barrier (+2 kcal mol^−1^). The solid-state rotational motions reached a non-Brownian, inertial regime at 335 K (Matsuno et al., 2018b).

Unlike the relatively rigid conformation of the arylene panels in [4]CC, [7]cyclo-amphi-naphthylene ([7]CaNAP) was rather flexible with its panels rotate rapidly at ambient temperature (Sun et al., 2016). However, this rotation did not significantly affect its binding ability for C_70_ with the *K*_a_ in the range of 10^7^-10^9^ M^−1^ (depending on the solvents) (Figure 1O) (Sun et al., 2019). More importantly, the structure of [7]CaNAP deformed during the rotation to track the orientation changes of the ellipsoidal C_70_.

By using dumbbell-shaped C_60_ dimer (C_120_) as the ligand with two binding sites, two-wheeled composites can be assembled with the shape-persistent macrocycles as the receptors (Matsuno et al., 2016, 2017). The thermodynamics of the 2:1 complex revealed the two-stage association constants, for example *K*_a1_ of 7.3 × 10^11^ M^−1^ for the formation of the 1:1 complex [(*P*)-(12,8)-[4]CC⊃C_120_], and *K*_a2_ of 9.7 × 10^7^ M^−1^ for the 2:1 complex [(*P*)-(12,8)-[4]CC⊃C_120_⊂(*P*)-(12,8)-[4]CC]. There was no self-assembly of the two [4]CC hosts without C_120_. The ligand-induced self-sorting phenomena was observed from the [4]CC family⊃C_120_. A moderate level of self-sorting was obtained when mixing a racemic mixture ([4]CC ((*P*)-(12,8)-[4]CC [(*P*)-*D*_4_] and (*M*)-(12,8)-[4]CC [(*M*)-*D*_4_]) and C_120_ with equivalent molar ratio: yielding 70% amount of the racemate complexes [(*P*)-*D*_4_⊃C_120_⊂(*P*)-*D*_4_ + (*M*)-*D*_4_⊃C_120_⊂(*M*)-*D*_4_], and 30% amount of the meso-form [(*P*)-*D*_4_⊃C_120_⊂(*M*)-*D*_4_]. A complete self-sorting was obtained when two diastereomers of [4]CC ([(*P*)-*D*_4_] and (10,10)_ABAB_-[4]CC [*D*_2d_]) were applied: yielding 50% amount of (*P*)-*D*_4_⊃C_120_⊂(*P*)-*D*_4_, 50% amount of *D*_2d_⊃C_120_⊂*D*_2d_, and no (*P*)-*D*_4_⊃C_120_⊂*D*_2d_ was detected (Figure 1P). This shape recognition can be explained by the repulsive van der Waals interactions between aliphatic side chains caused by the H–H contacts at the interfaces of the receptors as revealed by the crystal structures.

## Supramolecular Complexes With Non-fullerene Compounds

When two aromatic moieties stack in a face-to-face fashion, the π-π interaction could hold the two species together, such as the case of CPP analogs with fullerenes. Besides, other non-covalent interactions, such as CH-π, metal-π interactions also play important roles in various supramolecular systems. The CH-π interaction, which is a kind of atom-to-surface hydrogen bond and relatively weak, could also assemble host-guest complex. On the other hand, the metal-π coordination usually could strongly stabilize the associated architecture.

**Figure 2:**
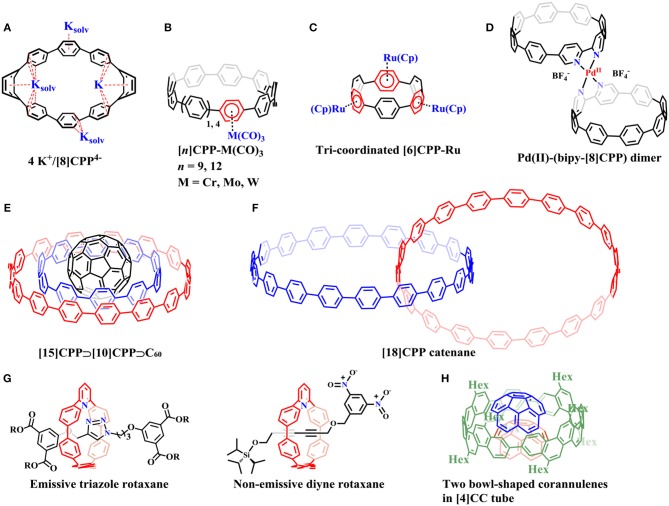
Supramolecular complexes with non-fullerene compounds: **(A)** The potassium salt of a CPP tetraanion: 4 K^+^/[8]${\text{CPP}}_{.}^{4-}$ **(B)** The η^6^ mono-coordinated CPP complexes. **(C)** Tri-coordinated complex of ruthenium with [6]CPP. **(D)** Pd(II) coordinated with two 2,2′-bipyridine-embedded [8]CPPs (bipy-[8]CPP) to form (bipy-[8]CPP)-Pd(II)-(bipy-[8]CPP) complex. **(E)** A ternary complex: [15]CPP⊃[10]CPP⊃C_60._ **(F)** [18]CPP catenane. **(G)** Emissive triazole rotaxanes and non-emissive diyne rotaxane. **(H)** The bowl-shaped corannulenes were encapsulated by [4]CC host through multiple weak CH-π contacts.

In 2013, Petrukhina et al. reported the potassium salt of a CPP tetraanion (4 K^+^/[8]CPP^4−^) by direct reduction of [8]CPP with potassium metal (Figure 2A) (Zabula et al., 2013). The X-ray diffraction analysis revealed that [8]CPP^4−^ functions as a multisite ligand with its *endo*- and *exo*- surfaces engaged in coordination with the potassium. Similarly, [6]CPP were also demonstrated to be reduced by alkali-metal to its mono- and di-anions, [6]CPP^1−^ and [6]CPP^2−^ (Spisak et al., 2018). Itami et al. synthesized η^6^ mono-coordinated CPP complexes [*n*]CPP-M(CO)_3_ where *n* = 9, 12 and M = Cr, Mo, W (Figure 2B) (Kubota et al., 2015). The crystal structure of [9]CPP-Cr(CO)_3_ showed that chromium coordinated on the convex surface of [9]CPP. Later, Yamago's group succeeded in the preparation of mono-, di-, and tri-coordinated complexes of ruthenium with [*n*]CPP (*n* = 5 and 6) (Kayahara et al., 2016). Ru selectively coordinated to alternate phenylene units in multi-coordinated complexes (Figure 2C). Single-crystal analysis indicated that Ru also coordinated on the convex surface of CPPs. More recently, Jasti illustrated a general strategy for building up nanohoops that could easily coordinate to transition metals (Van Raden et al., 2017). 2,2′-bipyridine-embedded [8]CPP (bipy-[8]CPP) synthesized in this work can readily coordinate to Pd(II) or Ru(II) metal centers, forming (bipy-[8]CPP)-Pd(II)-(bipy-[8]CPP) (Figure 2D) or Ru(II)-(bipy-[8]CPP) complexes, respectively.

Besides the role as supramolecular hosts, CPP molecules can also serve as guests to be included in larger nanohoops with the “Russian doll” fashion. The strongest binding was predicted when the host and guest differed by five phenyl rings through theoretical calculations (Fomine et al., 2012; Bachrach and Zayat, 2016). Yamago et al. demonstrated these predictions by experimental studies: [*n*]CPPs (*n* = 5, 6, 7, 8, and 10) did selectively interact with [*n*+5]CPPs, forming [*n*+5]CPP⊃[*n*]CPP complexes (Hashimoto et al., 2017). A ternary complex, [15]CPP⊃[10]CPP⊃C_60_, could also be assembled (Figure 2E).

By analyzing the ions in the gas phase of the complex mixture from CPP synthesis through matrix assisted laser desorption ionization (MALDI) together with ion-mobility mass spectrometry (IMMS), Müllen's group provided evidence for the existence of possible catenanes composed of CPPs, such as [12]CPP+[24]CPP, 2 × [18]CPP (Figure 2F), or even a trefoil knot (Zhang et al., 2017b). Most recently, Itami et al. reported the synthesis of all-benzene catenanes and trefoil knot through silicon-based template method which adjoined two neighboring CPP fragments in a crossing pattern followed by removal of the silicon tether after macrocyclization (Segawa et al., 2019). Interestingly, the trefoil knot shows only a single proton resonance in ^1^H-NMR spectrum even at −95°C, indicating its ultrafast motion on the NMR time scale. The [2]heterocatenane, in which [12]CPP and [9]CPP are mechanically interlocked shows energy transfer from [12]CPP to [9]CPP *via* the mechanical bond under light irradiation. Cong et al. reported the synthesis of a catenane consisting of two interlocked phenanthroline-containing nanohoops by copper(I)-templated method (Fan et al., 2018). The solid state structure shows a Möbius topology stabilized by non-covalentinteractions. A 2,6-pyridyl embedded nanohoops were recently synthesized for the preparation of nanohoop-based rotaxanes through active metal template reactions (Figure 2G; Van Raden et al., 2019). The triazole-embedded [2]rotaxanes showed dramatic changes in fluorescence emission (turn-off) when Pd(II) salt was added, suggesting its possible applications in ion sensing. Inspired by this study, another non-emissive [2]rotaxane was devised and synthesized, which has a fluorescence-quenching 3,5-dinitrobenzyl stopper and a fluoride-cleavable triisopropylsilyl (TIPS) stopper. Upon the addition of tetra-*n*-butylammonium fluoride (TBAF), 123-fold emission was recovered as the nanohoop fluorophore was released, indicating that the nanohoop rotaxanes could effectively serve as turn-on fluorescence sensors.

Itami et al. described the assembly of iodine within [*n*]CPPs (*n* = 9, 10, and 12) (Ozaki et al., 2017). Upon electric stimuli, [10]CPP-I turned out to emit white light, caused by the formation of polyiodide chains inside the [10]CPP cavity through charge transfer between [10]CPP tubes and encapsulated iodine chains.

Gaeta reported the 1,4-dimethoxy modified [8]CPP which exhibits binding ability toward pyridinium cations (Della Sala et al., 2017). Density functional theory (DFT) calculations indicated that the CH···π and N^+^···π^DMB^ (DMB = 1,4-dimethoxybenzene) interactions between the host and pyridinium guest played a crucial role in this supramolecular system. Another multi-(1,4-dimethoxy) modified [9]CPP synthesized by our group showed only weak supramolecular interactions for cationic molecules (Lu et al., 2018).

A novel type of host-guest complex assembled solely by CH-π hydrogen bonds rather than π-π interactions was devised by the Isobe group (Matsuno et al., 2018a). A bowl-shaped corannulene can be encapsulated by a [4]CC host through multiple weak CH-π contacts to form a 1:1 complex in solution, driven by a large association enthalpy. The 1:2 host-guest combination was unveiled in the crystalline solid state (Figure 2H). Despite the multiple weak hydrogen bonds, the guest was still allowed dynamic rotational motions in the host. Solid state analysis revealed a single-axis rotation of the bowl in the tube.

## Summary and Outlook

In this featured article, we overviewed recent progress on supramolecular properties of CPPs and their analogs. Various types of new carbon nanohoops were prepared by transition metal-catalyzed coupling reactions. These macrocycles usually possess well-defined cavities with rigid conformation and fixed diameters, which makes them good supramolecular hosts for incorporating a wide range of compounds, such as spherical fullerenes through π-π, metal-π, and/or CH-π interactions. These non-covalent interactions enabled efficient molecular recognitions and host-guest energy transfer. Although the synthesis of new carbon nanohoops and related supramolecular complexes has been growing very fast during the past decade, the applications of these carbon-rich architectures in some fields, such as, organic electronic devices, molecular sensing, and molecular machines, is still far from satisfaction. For further advancement, research efforts should be devoted to explore robust synthetic strategies which are essential for the diversification of carbon nanohoop family. Interdisciplinary studies with cooperative material sciences, analytical, biological, physical, and theoretical chemistry, will dramatically expand the understanding and application of the macrocycles and their supramolecular complexes. It is reasonable to expect that these carbon-rich structures will attract further research interests, and lead to the preparation of unique and unprecedented molecular tools and materials in the future.

## Author Contributions

PD supervised the project. DL and PD mainly wrote the paper. QH, SW, JW, and PH co-wrote the paper. All authors discussed the results and commented on the manuscript.

### Conflict of Interest

The authors declare that the research was conducted in the absence of any commercial or financial relationships that could be construed as a potential conflict of interest.

## References

[B1] Bachrach S. M. Zayat Z. C. (2016). “Planetary orbit” systems composed of cycloparaphenylenes. J. Org. Chem. 81, 4559–4565. 10.1021/acs.joc.6b00339 27163409

[B2] Cui S. Zhuang G. Lu D. Huang Q. Jia H. Wang Y.. (2018). A three-dimensional capsule-like carbon nanocage as a segment model of capped zigzag [12,0] carbon nanotubes: synthesis, characterization, and complexation with C_70_. Angew. Chem. Int. Ed. 57, 9330–9335. 10.1002/anie.201804031 29771461

[B3] Darzi E. R. Hirst E. S. Weber C. D. Zakharov L. N. Lonergan M. C. Jasti R. (2015). Synthesis, properties, and design principles of donor-acceptor nanohoops. ACS Cent. Sci. 1, 335–342. 10.1021/acscentsci.5b00269 27162989 PMC4827663

[B4] Della Sala P. Talotta C. Caruso T. De Rosa M. Soriente A. Neri P.. (2017). Tuning cycloparaphenylene host properties by chemical modification. J. Org. Chem. 82, 9885–9889. 10.1021/acs.joc.7b01588 28805382

[B5] Dong R. Zhou Y. Huang X. Zhu X. Lu Y. Shen J. (2015). Functional supramolecular polymers for biomedical applications. Adv. Mater. 27, 498–526. 10.1002/adma.201402975 25393728

[B6] Fan Y. Y. Chen D. Huang Z. A. Zhu J. Tung C. H. Wu L. Z.. (2018). An isolable catenane consisting of two Möbius conjugated nanohoops. Nat. Commun. 9:3037. 10.1038/s41467-018-05498-6 30072717 PMC6072741

[B7] Fomine S. Zolotukhin M. G. Guadarrama P. (2012). “Russian doll” complexes of [n]cycloparaphenylenes: a theoretical study. J. Mol. Model. 18, 4025–4032. 10.1007/s00894-012-1402-7 22460523

[B8] Hashimoto S. Iwamoto T. Kurachi D. Kayahara E. Yamago S. (2017). Shortest double-walled carbon nanotubes composed of cycloparaphenylenes. Chem Plus Chem 82, 1015–1020. 10.1002/cplu.201700097 31961607

[B9] Hitosugi S. Iizuka R. Yamasaki T. Zhang R. Murata Y. Isobe H. (2013). Assessment of fullerene derivatives as rolling journals in a finite carbon nanotube bearing. Org. Lett. 15, 3199–3201. 10.1021/ol400982r 23795989

[B10] Hitosugi S. Nakanishi W. Yamasaki T. Isobe H. (2011). Bottom-up synthesis of finite models of helical (n,m)-single-wall carbon nanotubes. Nat. Commun. 2, 492 10.1038/ncomms1505

[B11] Hitosugi S. Ohkubo K. Iizuka R. Kawashima Y. Nakamura K. Sato S.. (2014). Photoinduced electron transfer in a dynamic supramolecular system with curved π-structures. Org. Lett. 16, 3352–3355. 10.1021/ol501381x 24918189

[B12] Hitosugi S. Ohkubo K. Kawashima Y. Matsuno T. Kamata S. Nakamura K.. (2015). Modulation of energy conversion processes in carbonaceous molecular bearings. Chem. Asian J. 10, 2404–2410. 10.1002/asia.201500673 26195132

[B13] Huang Q. Zhuang G. Jia H. Qian M. Cui S. Yang S.. (2019). Photoconductive curved-nanographene/fullerene supramolecular heterojunctions. Angew. Chem. Int. Ed. 58, 6244–6249. 10.1002/anie.201900084 30843633

[B14] Huang Z. A. Chen C. Yang X. D. Fan X. B. Zhou W. Tung C. H.. (2016). Synthesis of oligoparaphenylene-derived nanohoops employing an anthracene photodimerization-cycloreversion strategy. J. Am. Chem. Soc. 138, 11144–11147. 10.1021/jacs.6b07673 27539737

[B15] Isobe H. Hitosugi S. Yamasaki T. Iizuka R. (2013). Molecular bearings of finite carbon nanotubes and fullerenes in ensemble rolling motion. Chem. Sci. 4, 1293–1297. 10.1039/c3sc22181d

[B16] Isobe H. Nakamura K. Hitosugi S. Sato S. Tokoyama H. Yamakado H.. (2015). Theoretical studies on a carbonaceous molecular bearing: association thermodynamics and dual-mode rolling dynamics. Chem. Sci. 6, 2746–2753. 10.1039/C5SC00335K 29142679 PMC5654412

[B17] Iwamoto T. Slanina Z. Mizorogi N. Guo J. Akasaka T. Nagase S.. (2014). Partial charge transfer in the shortest possible metallofullerene peapod, La@C_82_⊂[11]cycloparaphenylene. Chem. Eur. J. 20, 14403–14409. 10.1002/chem.201403879 25224281

[B18] Iwamoto T. Watanabe Y. Sadahiro T. Haino T. Yamago S. (2011). Size-selective encapsulation of C_60_ by [10]cycloparaphenylene: formation of the shortest fullerene-peapod. Angew. Chem. Int. Ed. 50, 8342–8344. 10.1002/anie.201102302 21770005

[B19] Iwamoto T. Watanabe Y. Takaya H. Haino T. Yasuda N. Yamago S. (2013). Size- and orientation-selective encapsulation of C_70_ by cycloparaphenylenes. Chem. Eur. J. 19, 14061–14068. 10.1002/chem.201302694 24108598

[B20] Jasti R. Bertozzi C. R. (2010). Progress and challenges for the bottom-up synthesis of carbon nanotubes with discrete chirality. Chem. Phys. Lett. 494, 1–7. 10.1016/j.cplett.2010.04.067 21224898 PMC2942765

[B21] Jasti R. Bhattacharjee J. Neaton J. B. Bertozzi C. R. (2008). Synthesis, characterization, and theory of [9]-, [12]-, and [18]cycloparaphenylene: carbon nanohoop structures. J. Am. Chem. Soc. 130, 17646–17647. 10.1021/ja807126u 19055403 PMC2709987

[B22] Kawase T. Darabi H. R. Oda M. (1996). Cyclic [6]- and [8]Paraphenylacetylenes. Angew. Chem. Int. Ed. 35, 2664–2666. 10.1002/anie.199626641

[B23] Kawase T. Nishiyama Y. Nakamura T. Ebi T. Matsumoto K. Kurata H.. (2007). Cyclic [5]paraphenyleneacetylene: synthesis, properties, and formation of a ring-in-ring complex showing a considerably large association constant and entropy effect. Angew. Chem. Int. Ed. 46, 1086–1088. 10.1002/anie.200603707 17183516

[B24] Kawase T. Tanaka K. Fujiwara N. Darabi H. R. Oda M. (2003a). Complexation of a carbon nanoring with fullerenes. Angew. Chem. Int. Ed. 42, 1624–1628. 10.1002/anie.200250728 12698460

[B25] Kawase T. Tanaka K. Seirai Y. Shiono N. Oda M. (2003b). Complexation of carbon nanorings with fullerenes: supramolecular dynamics and structural tuning for a fullerene sensor. Angew. Chem. Int. Ed. 42, 5597–5600. 10.1002/anie.200352033 14639725

[B26] Kayahara E. Patel V. K. Mercier A. Kundig E. P. Yamago S. (2016). Regioselective synthesis and characterization of multinuclear convex-bound ruthenium-[n]cycloparaphenylene (n = 5 and 6) complexes. Angew. Chem. Int. Ed. 55, 302–306. 10.1002/anie.201508003 26494105

[B27] Kubota N. Segawa Y. Itami K. (2015). η^6^-Cycloparaphenylene transition metal complexes: synthesis, structure, photophysical properties, and application to the selective monofunctionalization of cycloparaphenylenes. J. Am. Chem. Soc. 137, 1356–1361. 10.1021/ja512271p 25580526

[B28] Lehn J. M. (1985). Supramolecular chemistry: receptors, catalysts, and carriers. Science 227, 849–856. 10.1126/science.227.4689.849 17821215

[B29] Lehn J. M. (1988). Supramolecular chemistry-scope and perspectives molecules, supermolecules, and molecular devices. Angew. Chem. Int. Ed. 27, 89–112. 10.1002/anie.198800891

[B30] Lu D. Wu H. Dai Y. Shi H. Shao X. Yang S.. (2016). A cycloparaphenylene nanoring with graphenic hexabenzocoronene sidewalls. Chem. Commun. 52, 7164–7167. 10.1039/C6CC03002E 27172905

[B31] Lu D. Zhuang G. Wu H. Wang S. Yang S. Du P. W. (2017). A large π-extended carbon nanoring based on nanographene units: bottom-up synthesis, photophysical properties, and selective complexation with fullerene C_70_. Angew. Chem. Int. Ed. 56, 158–162. 10.1002/ange.201608963 27910250

[B32] Lu D. P. Zhuang G. L. Jia H. X. Wang J. Y. Huang Q. Cui S. S. (2018). A novel symmetrically multifunctionalized dodecamethoxy-cycloparaphenylene: synthesis, photophysical, and supramolecular properties. Org. Chem. Front. 5, 1446–1451. 10.1039/C8QO00033F

[B33] Matsuno T. Fujita M. Fukunaga K. Sato S. Isobe H. (2018a). Concyclic CH-π arrays for single-axis rotations of a bowl in a tube. Nat. Commun. 9:3779. 10.1038/s41467-018-06270-6 30224711 PMC6141547

[B34] Matsuno T. Kamata S. Hitosugi S. Isobe H. (2013). Bottom-up synthesis and structures of π-lengthened tubular macrocycles. Chem. Sci. 4:3179 10.1039/c3sc50645b

[B35] Matsuno T. Kamata S. Sato S. Yokoyama A. Sarkar P. Isobe H. (2017). Assembly, thermodynamics, and structure of a two-wheeled composite of a dumbbell-shaped molecule and cylindrical molecules with different edges. Angew. Chem. Int. Ed. 56, 15020–15024. 10.1002/anie.201709442 28994189

[B36] Matsuno T. Nakai Y. Sato S. Maniwa Y. Isobe H. (2018b). Ratchet-free solid-state inertial rotation of a guest ball in a tight tubular host. Nat. Commun. 9:1907. 10.1038/s41467-018-04325-2 29765050 PMC5954156

[B37] Matsuno T. Sato S. Iizuka R. Isobe H. (2015). Molecular recognition in curved π-systems: effects of π-lengthening of tubular molecules on thermodynamics and structures. Chem. Sci. 6, 909–916. 10.1039/C4SC02812K 29560176 PMC5811110

[B38] Matsuno T. Sato S. Yokoyama A. Kamata S. Isobe H. (2016). Self-sorting of two hydrocarbon receptors with one carbonaceous ligand. Angew. Chem. Int. Ed. 55, 15339–15343. 10.1002/anie.201609444 27865043

[B39] Miki K. Matsushita T. Inoue Y. Senda Y. Kowada T. Ohe K. (2013). Electron-rich carbon nanorings as macrocyclic hosts for fullerenes. Chem. Commun. 49, 9092–9094. 10.1039/c3cc42561d 23715441

[B40] Nakanishi Y. Omachi H. Matsuura S. Miyata Y. Kitaura R. Segawa Y.. (2014). Size-selective complexation and extraction of endohedral metallofullerenes with cycloparaphenylene. Angew. Chem. Int. Ed. 53, 3102–3106. 10.1002/anie.201311268 24616170

[B41] Ogoshi T. Kanai S. Fujinami S. Yamagishi T. A. Nakamoto Y. (2008). *para*-Bridged symmetrical pillar[5]arenes: their Lewis acid catalyzed synthesis and host-guest property. J. Am. Chem. Soc. 130, 5022–5023. 10.1021/ja711260m 18357989

[B42] Ozaki N. Sakamoto H. Nishihara T. Fujimori T. Hijikata Y. Kimura R.. (2017). Electrically activated conductivity and white light emission of a hydrocarbon nanoring-iodine assembly. Angew. Chem. Int. Ed. 56, 11196–11202. 10.1002/anie.201703648 28585773

[B43] Povie G. Segawa Y. Nishihara T. Miyauchi Y. Itami K. (2017). Synthesis of a carbon nanobelt. Science 356, 172–175. 10.1126/science.aam8158 28408599

[B44] Povie G. Segawa Y. Nishihara T. Miyauchi Y. Itami K. (2018). Synthesis and size-dependent properties of [12], [16], and [24]Carbon nanobelts. J. Am. Chem. Soc. 140, 10054–10059. 10.1021/jacs.8b06842 30032597

[B45] Quernheim M. Golling F. E. Zhang W. Wagner M. Räder H. J. Nishiuchi T.. (2015). The precise synthesis of phenylene-extended cyclic hexa-peri-hexabenzocoronenes from polyarylated [n]cycloparaphenylenes by the scholl reaction. Angew. Chem. Int. Ed. 54, 10341–10346. 10.1002/anie.201500392 26110414

[B46] Rio J. Beeck S. Rotas G. Ahles S. Jacquemin D. Tagmatarchis N. (2018). Electronic communication between two [10]cycloparaphenylenes and bis(azafullerene) (C_59_N)_2_ induced by cooperative complexation. Angew. Chem. Int. Ed. 57, 6930–6934. 10.1002/anie.201713197 29573077

[B47] Sato S. Yamasaki T. Isobe H. (2014). Solid-state structures of peapod bearings composed of finite single-wall carbon nanotube and fullerene molecules. Proc. Natl. Acad. Sci. U.S.A. 111, 8374–8379. 10.1073/pnas.1406518111 24912184 PMC4060721

[B48] Segawa Y. Fukazawa A. Matsuura S. Omachi H. Yamaguchi S. Irle S.. (2012). Combined experimental and theoretical studies on the photophysical properties of cycloparaphenylenes. Org. Biomol. Chem. 10, 5979–5984. 10.1039/c2ob25199j 22441238

[B49] Segawa Y. Kuwayama M. Hijikata Y. Fushimi M. Nishihara T. Pirillo J.. (2019). Topological molecular nanocarbons: All-benzene catenane and trefoil knot. Science 365, 272–276. 10.1126/science.aav5021 31320538

[B50] Segawa Y. Yagi A. Matsui K. Itami K. (2016). Design and synthesis of carbon nanotube segments. Angew. Chem. Int. Ed. 55, 5136–5158. 10.1002/anie.201508384 26890967

[B51] Spisak S. N. Wei Z. Darzi E. Jasti R. Petrukhina M. A. (2018). Highly strained [6]cycloparaphenylene: crystallization of an unsolvated polymorph and the first mono- and dianions. Chem. Commun. 54, 7818–7821. 10.1039/C8CC03693D 29946580

[B52] Sun Z. Mio T. Okada T. Matsuno T. Sato S. Kono H.. (2019). Unbiased rotational motions of an ellipsoidal guest in a tight yet pliable host. Angew. Chem. Int. Ed. 58, 2040–2044. 10.1002/anie.201812771 30549181

[B53] Sun Z. Suenaga T. Sarkar P. Sato S. Kotani M. Isobe H. (2016). Stereoisomerism, crystal structures, and dynamics of belt-shaped cyclonaphthylenes. Proc. Natl. Acad. Sci. U.S.A. 8109–8114. 10.1073/pnas.1606530113 27357686 PMC4961134

[B54] Toyota S. Tsurumaki E. (2019). Exploration of nano-saturns: a spectacular sphere-ring supramolecular system. Chem. Eur. J. 25, 6878–6890. 10.1002/chem.201900039 30688383

[B55] Ueno H. Nishihara T. Segawa Y. Itami K. (2015). Cycloparaphenylene-based ionic donor-acceptor supramolecule: isolation and characterization of Li^+^@C_60_⊂[10]CPP. Angew. Chem. Int. Ed. 54, 3707–3711. 10.1002/anie.201500544 25693784

[B56] Van Raden J. M. Louie S. Zakharov L. N. Jasti R. (2017). 2,2′-Bipyridyl-embedded cycloparaphenylenes as a general strategy to investigate nanohoop-based coordination complexes. J. Am. Chem. Soc. 139, 2936–2939. 10.1021/jacs.7b00359 28212009

[B57] Van Raden J. M. White B. M. Zakharov L. N. Jasti R. (2019). Nanohoop rotaxanes from active metal template syntheses and their potential in sensing applications. Angew. Chem. Int. Ed. 58, 7341–7345. 10.1002/anie.201901984 30913355

[B58] Wu D. Cheng W. Ban X. T. Xia J. L. (2018). Cycloparaphenylenes (CPPs): an overview of synthesis, properties, and potential applications. Asian, J. Org. Chem. 7, 2161–2181. 10.1002/ajoc.201800397

[B59] Xia J. Bacon J. W. Jasti R. (2012). Gram-scale synthesis and crystal structures of [8]- and [10]CPP, and the solid-state structure of C_60_@[10]CPP. Chem. Sci. 3, 3018–3021. 10.1039/c2sc20719b

[B60] Xu Y. Delius M. (2019). The supramolecular chemistry of strained carbon nanohoops. Angew. Chem. Int. Ed. 10.1002/anie.201906069. [Epub ahead of print]. 31190449

[B61] Xu Y. Kaur R. Wang B. Minameyer M. B. Gsanger S. Meyer B. (2018a). Concave-convex π-π template approach enables the synthesis of [10]cycloparaphenylene-fullerene [2]rotaxanes. J. Am. Chem. Soc. 140, 13413–13420. 10.1021/jacs.8b08244 30234982

[B62] Xu Y. Wang B. Kaur R. Minameyer M. B. Bothe M. Drewello T.. (2018b). A supramolecular [10]CPP junction enables efficient electron transfer in modular porphyrin-[10]CPP⊃fullerene complexes. Angew. Chem. Int. Ed. 57, 11549–11553. 10.1002/anie.201802443 29985554

[B63] Yan X. Wang F. Zheng B. Huang F. (2012). Stimuli-responsive supramolecular polymeric materials. Chem. Soc. Rev. 41, 6042–6065. 10.1039/c2cs35091b 22618080

[B64] Yang L. Tan X. Wang Z. Zhang X. (2015). Supramolecular polymers: historical development, preparation, characterization, and functions. Chem. Rev. 115, 7196–7239. 10.1021/cr500633b 25768045

[B65] Yuan K. Guo Y. J. Zhao X. (2015). Nature of noncovalent interactions in the [n]cycloparaphenylene⊃C_70_ (n = 10, 11, and 12) host–guest complexes: a theoretical insight into the shortest C_70_-carbon nanotube peapod. J. Phys. Chem. C 119, 5168–5179. 10.1021/jp5129657

[B66] Zabula A. V. Filatov A. S. Xia J. Jasti R. Petrukhina M. A. (2013). Tightening of the nanobelt upon multielectron reduction. Angew. Chem. Int. Ed. 52, 5033–5036. 10.1002/anie.201301226 23564669

[B67] Zhang D. Martinez A. Dutasta J. P. (2017a). Emergence of hemicryptophanes: from synthesis to applications for recognition, molecular machines, and supramolecular catalysis. Chem. Rev. 117, 4900–4942. 10.1021/acs.chemrev.6b00847 28277650

[B68] Zhang W. Abdulkarim A. Golling F. E. Räder H. J. Müllen K. (2017b). Cycloparaphenylenes and their catenanes: complex macrocycles unveiled by ion mobility mass spectrometry. Angew. Chem. Int. Ed. 56, 2645–2648. 10.1002/anie.201611943 28146311

[B69] Zhang X. Wang C. (2011). Supramolecular amphiphiles. Chem. Soc. Rev. 40, 94–101. 10.1039/B919678C 20890490

[B70] Zhou J. Yu G. Huang F. (2017). Supramolecular chemotherapy based on host-guest molecular recognition: a novel strategy in the battle against cancer with a bright future. Chem. Soc. Rev. 46, 7021–7053. 10.1039/C6CS00898D 28980674

